# Generation and characterization of a monoclonal antibody against an African swine fever virus protein encoded by the *A137R* gene

**DOI:** 10.3389/fvets.2023.1286906

**Published:** 2023-10-19

**Authors:** Carissa Embury-Hyatt, Estella Moffat, Dmytro Zhmendak, Cassidy N. G. Erdelyan, Brad Collignon, Kalhari Goonewardene, Aruna Ambagala, Ming Yang

**Affiliations:** National Centre for Foreign Animal Disease, Winnipeg, MB, Canada

**Keywords:** African swine fever virus, monoclonal antibody, antibody binding epitope, double-antibody sandwich ELISA, immunohistochemistry, *in situ* hybridization

## Abstract

The ongoing African swine fever (ASF) pandemic continues to have a major impact on global pork production and trade. Since ASF cannot be distinguished from other swine hemorrhagic fevers clinically, ASF-specific laboratory diagnosis is critical. Thus ASF virus (ASFV)-specific monoclonal antibodies (mAbs) are critical for the development of laboratory diagnostics. In this study, we report one ASFV-specific mAb, F88ASF-55, that was generated and characterized. This mAb recognizes the ASFV *A137R*-encoded protein (pA137R). Epitope mapping results revealed a highly conserved linear epitope recognized by this mAb, corresponding to amino acids 111–125 of pA137R. We explored the potential use of this mAb in diagnostic applications. Using F88ASF-55 as the detection antibody, six ASFV strains were detected in an enzyme-linked immunosorbent assay (ELISA) with low background. In immunohistochemistry (IHC) assays, this mAb specifically recognized ASFV antigens in the submandibular lymph nodes of animals experimentally infected with different ASFV strains. Although not all ASFV genotypes were tested in this study, based on the conserved ASFV epitope targeted by F88ASF-55, it has the potential to detect multiple ASFV genotypes. In conclusion, this newly generated ASFV pA137R-specific mAb has potential value in ASF diagnostic tool development. It can be used in ELISA, IHC, and possibly-immunochromatographic strip assays for ASFV detection. It also suggests that pA137R may be a good target for diagnostic assays to detect ASFV infection.

## Introduction

African swine fever (ASF) is a lethal, viral hemorrhagic disease of domestic pigs, and first reported from East Africa in 1921. This ongoing uninterrupted global spread continues to have a major impact on global pork production and trade (1). ASF is caused by the African swine fever virus (ASFV), the only virus belonging to the *Asfarviridae* family in the genus *Asfivirus* (2, 3). ASFV is a large, structurally complex double stranded DNA virus with an icosahedral symmetry and both an inner and outer lipid envelope (3). The ASF viral particle consists of several concentric layers that contribute to its structure; starting from the nucleoid containing the nuclear material, which is surrounded by the thick protein core shell, and a surrounding inner lipid envelope that is finally enclosed by a capsid (4). ASFV is classified into 24 genotypes based on B646L, which encode structural protein p72. ASFV strains can be divided into eight serogroups based on antibody-mediated hemadsorption inhibition (5). So far, no vaccine or antiviral is commercially available (6). ASFV originated in sub-Saharan Africa, where it remains endemic. However, following the introduction to Georgia in 2007, ASFV subsequently spread to Russia and Europe (1). In 2018, China reported the first ASF outbreak; since then, 16 Asia-Pacific countries have thus far reported this lethal swine disease (7). ASFV genotype II is the current circulating pandemic strain causing outbreaks in Europe, the Russian Federation, South East Asia, the Dominican Republic and Haiti (1, 8, 9). In addition, the presence of genotype II ASFV was reported for the first time in Nigeria and West Africa recently (10).

Since ASF cannot be differentiated from other swine hemorrhagic fevers clinically or by postmortem examination, ASF-specific laboratory diagnostic tools are critical. The rapid and early detection of ASFV infection is one of the key components in controlling this disease. ASFV-specific monoclonal antibodies (mAb) are essential for developing laboratory diagnostic assays. Several ASFV-specific mAbs have been generated previously; however, all mAbs were generated from animals immunized with recombinant proteins such as p30 (11–13) or p72 (14, 15). Recombinant protein immunogens can be used to generate highly specific antibodies to detect a single protein. When whole viruses are used as immunogens, mAbs can be generated against various viral proteins. Therefore, the generated mAbs can have multiple applications in different immunoassays. In addition, epitopes may be conserved in sequences so that individual antibodies may have reactivity across a number of virus genotypes.

In this study, whole ASFV was used as an immunogen, and one mAb F88ASF-55 was generated and characterized. The epitope recognized by the mAb was identified using a peptide array. The diagnostic application of this mAb was also examined. The results indicated that F88ASF-55 can be used for detecting ASFV in immunoassays, such as ELISA and immunohistochemistry assays.

## Materials and methods

### Preparation of African swine fever virus

All ASFV strains used in this study are listed in Table 1. To generate the intact ASFV virus particle for mice immunization, ASFV Lisbon/61, adapted in African green monkey kidney cells (Vero-76), was used (16). Briefly, Vero-76 cells (ATCC) grown in T-75 cell culture flasks were infected with ASFV Lisbon/61 at a multiplicity of infection (MOI) of 0.1. The infected cultures were incubated at 37°C in a 5% CO_2_ incubator for 5–7 days until 90%–100% of the cells showed cytopathic effects. The flasks were then frozen overnight at −20°C, thawed the next day, and the contents were harvested and then sonicated for 30 s three times on ice. The resulting cell suspension was centrifuged at 3,600 × *g* for 10 min at 4°C to remove cellular debris. The supernatant was then ultra centrifuged (JA20 Beckmann rotor) at 8500 rpm for 6 h at 4°C. The resulting pellet was resuspended in DMEM, layered onto a 40% sucrose cushion, and centrifuged at 20,000 rpm (49,400 × *g*) for 45 min at 4°C using a Beckman SW-41Ti rotor. The pellet was resuspended in PBS and aliquoted into 2 mL cryovials. The vials were then immersed in a 70°C water bath for 60 min and subsequently exposed to gamma irradiation (5 million rad) to inactivate the virus. The treated ASFV were used for mouse immunization. Other ASF viruses used in this study were propagated and stored as previously described (17).

**Table 1:** African swine fever virus detection using the double sandwich ELISA and immunohistochemistry assay.

ASFV isolates	Genotype	DAS ELISA	IHC
Lisbon/61	I	+	NA
BA71V	I	+	NA
OURT 88/3	I	+	NA
Ghana Akuse/2020	I	+	NA
Estonia 2014	II	+	+
Armenia/07	II	+	NA
Georgia 2007/1	II	NA	+
Gasson (Kenya/50)	X	NA	+
Lillie SI 85/South Africa	XX	NA	+

### Generation of monoclonal antibodies

Monoclonal antibodies against ASFV were produced as previously described (18, 19). Briefly, female BALB/c mice were inoculated with irradiated and concentrated ASFV Lisbon/61 (~20 μg/mouse) in an equal volume of TiterMax Gold (TiterMax Inc., United States) subcutaneously. A total of six mice were immunized and boosted four times after the initial immunization. Three to 4 days before to fusion, mice were boosted with the same antigen in phosphate-buffered saline (PBS) by intravenous injection. Immunized spleen cells were then fused with myeloma cells (P3X63 Ag8.653). After 2 weeks, hybridoma supernatants were screened using an ELISA. The positive clones were subcloned using a limiting dilution method. The mAbs were isotyped using a mouse monoclonal antibody isotyping kit (Roche, Indianapolis, IN, United States).

### Double-antibody sandwich ELISA

Anti-ASFV porcine serum was collected 48 days post-inoculation (dpi) with ASFV (Estonia 2014). Microtitre plates (Nunc-Immunoplate Maxisorp, Roskilde, Denmark) were coated with polyclonal porcine anti-ASFV (Estonia 2014) serum diluted 1:5000 in a carbonate/bicarbonate buffer, pH 9.6 overnight at 4°C. After blocking in casein blocking buffer (Sigma-Aldrich, United States), ASF viruses (Table 1) were added. Concentrated and irradiated ASFV Lisbon/61 was used as an antigen for hybridoma screening. Hybridoma culture supernatants were then added. After incubation, horseradish peroxidase (HRP)-conjugated anti-mouse IgG (1:2000, Jackson ImmunoResearch Laboratories, West Grove, PA, United States) was added, followed by-3,3′,5,5′-Tetramethylbenzidine (TMB, Pierce Biotechnology, Inc. Rockford, Illinois, United States). After stopping, optical density (OD) was measured at 450 nm using an Emax microplate reader (Molecular Devices, San Jose, CA, United States). Each incubation step was 60 min at 37°C with gentle shaking, followed by five washes (PBS with 0.1% Tween 20).

### Western blot analysis of ASFV protein

Irradiated and concentrated ASFV (Lisbon/61) was separated on a 4%–12% NuPAGE Novex Bis-Tris gel (Invitrogen, Carlsbad, United States); separated protein bands were transferred to nitrocellulose (NC) membranes using the iBlot Gel Transfer Device (Invitrogen, Carlsbad, United States). Membranes were then blocked using Casein blocking buffer at room temperature for 1 h and probed with either positive pig serum or mAbs diluted in Casein blocking buffer. Following primary antibody incubation, blots were incubated with HRP-conjugated anti-swine or anti-mouse secondary antibody (1:2000; Jackson ImmunoResearch Laboratories, West Grove, United States) for 1 h at room temperature. Blots were developed using the substrate 3,3′-diaminobenzidine (Sigma-Aldrich, St. Lucia, United States).

### Monoclonal antibody binding protein identification using liquid chromatography-mass spectrometry

SDS-PAGE gel slices were de-stained using a combination of alternating washes with 100 mM ammonium bicarbonate and acetonitrile (Thermo Fisher Scientific, MA, United States). Once the Coomassie blue stain was removed, and the proteins within the gel slices were reduced and alkylated by the addition of 10 mM dithiothreitol and 66 mM iodoacetamide (Sigma Aldrich, MO, United States), respectively. The proteins within the gel slices were then digested using trypsin (Thermo Fisher Scientific, MA, United States), and the peptides were extracted from the gel slices using a combination of alternating washes with 1% formic acid (Millipore Sigma, MA, United States) and acetonitrile. The solution containing the tryptic peptides was dried to near completeness, and the peptides were resuspended in 2% acetonitrile, 0.1% formic acid (buffer A).

Digested peptide samples were analyzed using a nano-flow Easy nLC 1200 connected in-line to an Orbitrap Fusion Lumos mass spectrometer with a nano-electrospray ion source (2.3 kV) and a FAIMS Pro interface (Thermo Fisher Scientific, MA, United States). The samples were loaded (2 μL) onto a C18-reversed-phase Easy Spray column (50 cm long, 75 μm inner diameter, 2 μm particles, (Thermo Fisher Scientific) with 100% buffer A. Peptides were then eluted using a linear gradient of 3%–27% buffer B (80% acetonitrile, 0.1% formic acid) over 50 min, 27%–39% buffer B for 15 min, 39%–100% buffer B for 5 min and a wash at 100% buffer B for 10 min at a constant flow rate of 250 nL/min). Total liquid chromatography-mass spectrometry (LC-MS/MS) run-time was 100 min. The data-dependent acquisition was used with three different CV settings on the FAIMS Pro, (−40, −60, −80) 1 s each over a total cycle time of 3 s. The most abundant precursor ions in one-second survey scans were selectively isolated in the quadrupole (1.6 *m*/*z* isolation width) and fragmented by higher-energy collisional dissociation (HCD) (35% normalized collision energy). The survey scans were acquired in the Orbitrap over a range of *m*/*z* 375–1,500 with a target resolution of 120,000 at *m*/*z* 200, and the fragment ion scans were acquired in the linear ion trap with the scan rate set to “Rapid.”

Raw mass spectrometry data were converted to.mgf files using msconvert (ProteoWizard release 3.0.19254). Data was searched using Mascot (v3.1, Matrix Science) against a FASTA database of African Swine Fever Virus protein sequences and a database of common contaminant proteins. Mascot.dat files were imported into Scaffold (v 5.1.2) to generate a list of protein identifications.

### Peptide ELISA

A total of 14 overlapping peptides [15 amino acids (aa) in length, overlapping each other by 10 aa] were synthesized based on the ASFV *A137R*-encoded protein (pA137R) sequence (GenBank accession #AYW34014.1, ABclonal technology, MA, United States). Peptide ELISAs were performed as previously described (18, 19). Briefly, microtitre plates (Nunc Maxisorb) were coated with peptides (1 μg/well). After blocking, a diluted hybridoma culture supernatant was added to the plate. After incubation, HRP-conjugated anti-mouse IgG (1:2000, Jackson ImmunoResearch Laboratories, West Grove, PA, United States) was added. Then TMB was added and incubated for 15 min. After stopping, OD was measured at 450 nm using an Emax microplate reader. Each incubation step was 60 min at 37°C with gentle shaking, followed by five washes with a washing buffer.

### Tissues from ASFV-infected animals

Archived formalin-fixed, paraffin-embedded tissues were obtained from pigs experimentally inoculated with ASFV at the National Center for Foreign Animal Disease (NCFAD). Lymph nodes from animals infected with Gasson (7 dpi), Lillie SI-85 (5 dpi), Georgia 2007/1 (7 dpi), and Estonia 2014 (10 dpi). Tissues were sectioned at 5 μm onto positively charged slides for further testing in immunohistochemistry (IHC) and *in situ* hybridization (ISH) assays.

### Immunohistochemistry assay

Tissue sections were quenched for 10 min in aqueous 3% hydrogen peroxide. Epitopes were retrieved using an in-house glycan retrieval solution in a Biocare Medical Decloaking Chamber. Primary antibody F88ASF-55 was applied to sections at pre-titrated dilutions and incubated at room temperature for 30 min. Reactions were visualized using the HRP-conjugated anti-mouse polymer Envision^®^ + System (Agilent Technologies, CO, United States) and developed with the chromogenic reagent diaminobenzidine (DAB). Sections were then counterstained with Gill’s hematoxylin. Lymph nodes from uninfected animals were tested as negative controls in the IHC assay, and no immunostaining was observed.

### *In situ* hybridization

For the *in situ* hybridization (ISH) assay, the RNAScope 2.5 HD Detection Reagent-Red kit (Advanced Cell Diagnostics, Inc., CA, United States) was used. The procedure was performed following the manufacturer’s instructions. Briefly, tissue sections were cleared and hydrated in xylene and 100% ethanol and then air-dried. The sections were quenched for 10 min in aqueous H_2_O_2_, boiled in target retrieval solution for 15 min, rinsed in 100% ethanol and air-dried again. Then a final pre-treatment of protease plus enzyme for 15 min at 40°C was applied. The V-AFSV-01 probe (Advanced Cell Diagnostics, Inc., CA, United States) was applied and incubated at 40°C for 2 h. Sections were then washed twice in 1× wash buffer. After each of the subsequent hybridization steps, the sections were washed twice again. The signal was visualized with the chromogen Fast Red. The sections were then counterstained with Gill’s 1 hematoxylin, dried, cover-slipped with EcoMount (BioCare Medical, CA, United States), and examined by the pathologist. A negative control lymph node from a non-infected animal was tested in an ISH assay, and no immunostaining was observed. In addition, a negative control probe on positive tissue (lymph node from an ASFV Georgia 2007/1 infected animal) was tested, and no staining was observed.

## Results

### Generation of monoclonal antibodies against African swine fever virus

Following mouse immunization with concentrated ASFV Lisbon/61, splenocytes were harvested and fused with myeloma cells. Supernatants from the resulting hybridomas were screened using ASFV Lisbon/61 as the antigen in an ELISA. Positive clones were selected and then subcloned. To determine the specificity of the clones, subcloned hybridoma culture supernatants were further tested in an ELISA using foot-and-mouth disease virus and recombinant classical swine fever E2 protein as negative controls. Only one hybridoma clone, F88ASF-55, showed specificity for ASFV and no cross-reactivity with other antigens (data not shown). The F88ASF-55 mAb is an IgG1 isotype and contains a *ƙ* light chain.

### Determination of monoclonal antibody binding protein of African swine fever virus

**Figure 1:**
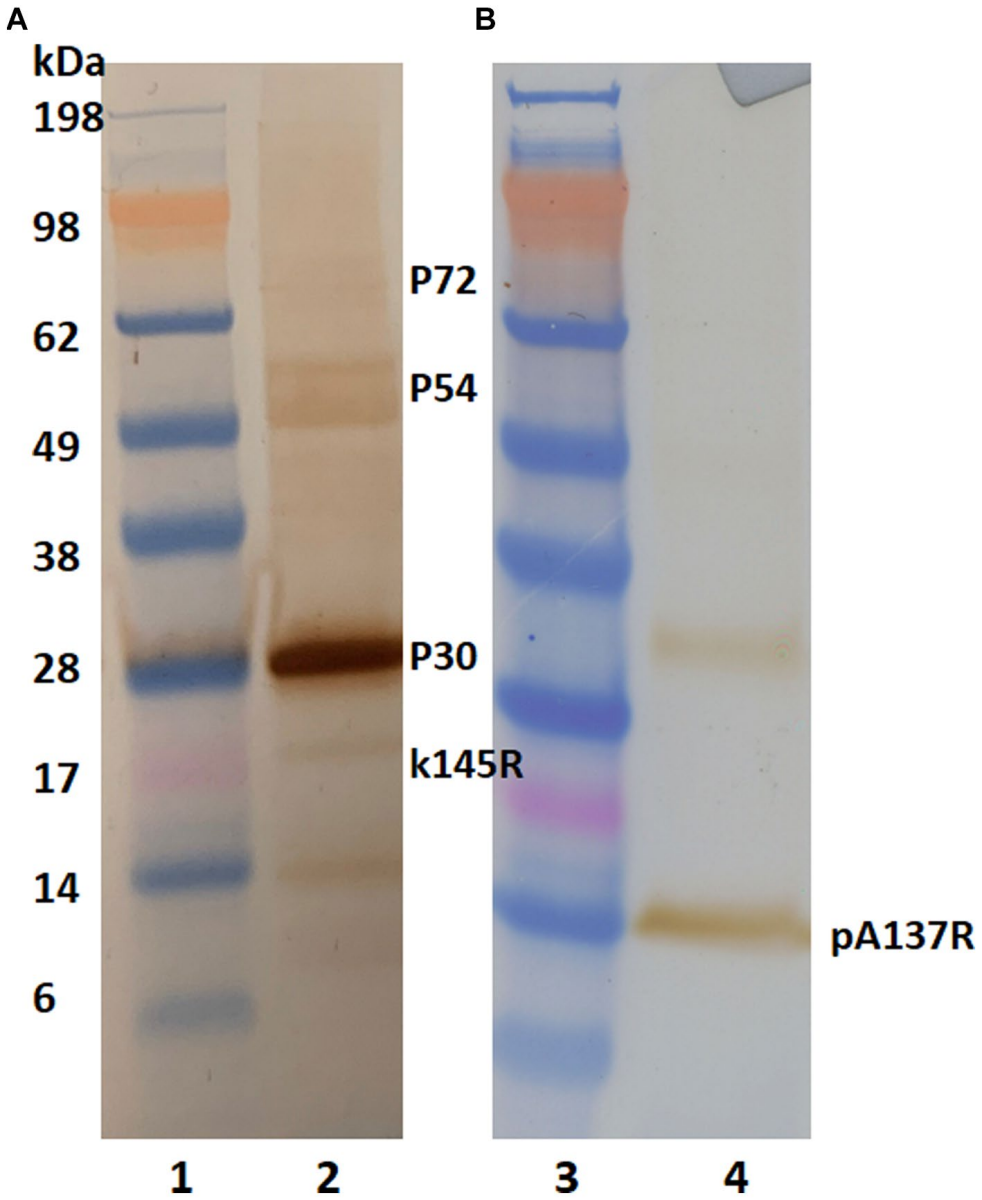
Antibody reactivity to ASFV structural proteins in western blot analysis. Concentrated African swine fever virus (Lisbon/61) proteins were separated on 4%–12% NuPAGE Novex Bis-Tris gels and transferred to nitrocellulose membranes. ASFV proteins were detected using an ASFV-positive serum **(A)** and mAb F88ASF-55 **(B)**. Blots were incubated with either HRP-conjugated anti-pig or HRP anti-mouse IgG. using 3, 3’–Diaminobenzidine was used for visualization. Lanes 1 and 3: MW marker; and lanes 2 and 4: ASFV protein.

To identify the mAb’s binding protein, western blot analysis was performed. Irradiated and concentrated ASFV Lisbon/61 was separated on NuPAGE Novex Bis-Tris gels and the proteins were transferred to nitrocellulose membranes. The ASFV proteins were detected with a positive pig serum (Figure 1A) and F88ASF-55 (Figure 1B). The ASFV-positive porcine serum detected ASFV proteins with molecular weights (MW) of approximately 30, 54, 72, and 17 kDa, corresponding to ASFV structural proteins p30, p54, p72, and k145Rp, respectively. The porcine polyclonal serum showed a strong reaction to p30, but was weak to p54, p72, and k145Rp (Figure 1A). Additionally, the polyclonal serum showed a weak reaction to a 14 kDa protein. While F88ASF-55 reacted strongly to a protein with a MW of 14 kDa and weakly to a protein with a MW of 33 kDa (Figure 1B). The epitope recognized by F88ASF-55 is linear because the mAb reacted with denatured ASFV protein in western blot analysis.

**Figure 2:**
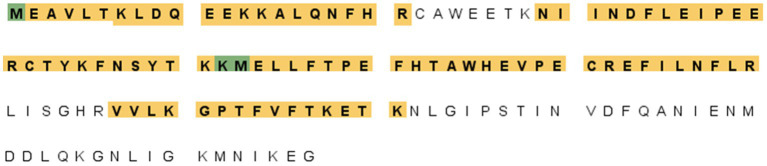
*A137R* protein (p11.5) sequence encoded by *A137R* with identified unique tryptic peptides. The protein bands (14 kDa) corresponding to the position indicated in the western blot were excised from SDS-PAGE, detained, digested with trypsin, and identified by peptide mass fingerprinting of ASFV structural proteins. The figure shows the amino acid sequence of the ASFV *A137R*-polyprotein. The amino acids highlighted were identified as unique tryptic peptides spanning the protein sequence. The amino acid coverage of the structural protein encoded by *A137R* was 64.2% (88/137).

A proteomic approach was performed to determine the sequence identities of the protein band (14 kDa) recognized by F88ASF-55. The stained protein band was excised, digested, and analyzed by LC-MS/MS. A total of 10 exclusive unique peptides and 15 exclusive unique spectra were identified (data not shown). The amino acid coverage (88/137) of the structural protein encoded by *A137R* was 64.2% (Figure 2). The results confirmed that the mAb-binding epitope is located on the protein encoded by *A137R* (formerly known as p11.5).

### Identification of mAb binding epitope

**Figure 3:**
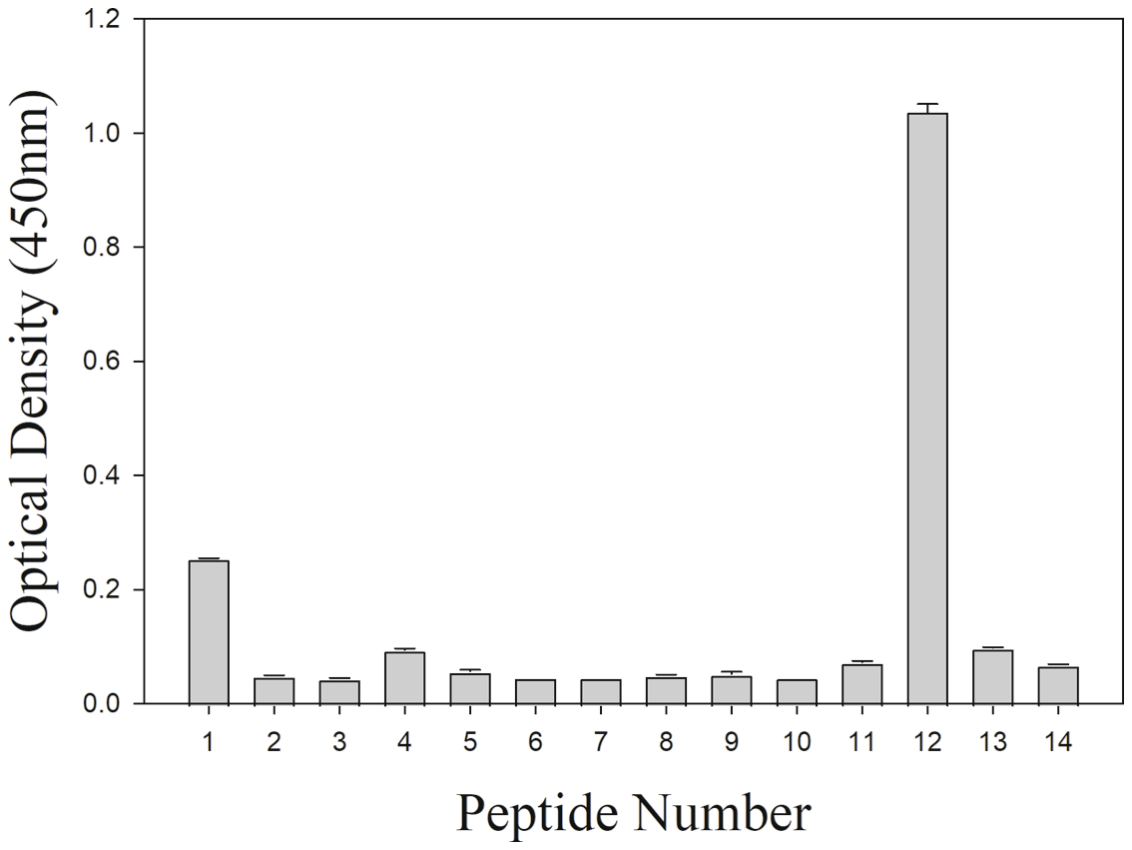
Peptide ELISA to ASFV protein peptides encoded by *A137R*. The mAb reactivity with pA137R peptides in ELISA. A total of 14 overlapping peptides [15 amino acids (aa) in length, overlapping by 10 aa] based on ASFV pA137R sequence were synthesized and coated onto a 96-well plate. Peptide binding by mAb F88ASF-55 was detected with a rabbit anti-mouse IgG-HRP conjugate. Optical density (OD) values are mean duplicates.

To further locate binding epitopes, 14 overlapping peptides representing the whole polypeptide encoded by *A137R* were synthesized. The reactivity of the mAb to ASFV pA137R peptides was examined using a peptide ELISA. The mAb reacted with peptide #12, corresponding to amino acids 111–125 (VDFQANIENMDDLQK) (Figure 3). Sequences of this region (111–125 aa) were aligned with ASFV isolates from 1950–2008 (Table 2). The results showed that the mAb-binding epitope was 100% identical across the ASFV 12 genotypes examined.

**Table 2:** Partial Amino acid sequence (74–137) alignment of African swine fever virus protein pA137R (p11.5) with twelve genotypes of ASFV isolates from 1950 to 2008.

Isolate	Genotype	Accession#	Amino acid sequence
E75	I	FN557520	80 90 100 111^a^ 125 137
			ILNFLRLISGHRVVLKGPTFVFTKETKNLGIPSTIN**VDFQANIENMDDLQK**GNLIGKMNIKEG
ASFV Georgia 2007/1	II	FR682468	.........................I......................................
Warmbaths	III	AY261365	..................I......I....................................S.
Warthog	IV	AY261366	..................II.....V....................................S.
Tengani 62	V	AY261364	..................II.....I......................................
Mkuzi 1979	VII	AY261362	................................................................
Malawi Lil-20/1 (1983)	VIII	AY261361	.I......T.........AI..........................................N.
Ken06.Bus	IX	KM111295	...Y...V.........SK.L.........................................S.
Kenya 1950	X	AY261360	...Y...V.........SSIL.........................................S.
TAN/08/Mazimbu	XV	ON409981	.I...K..T.........AI..........................................N.
Pretoriuskop/96/4	XX	AY261363	...................I.....I....................................S.
RSA_2_2008	XXII	MN336500	..................I......I....................................S.

a Epitope region of amino acids 111–125 (peptide #12) recognized by monoclonal antibody.

“·”Matching residues.

Although numerous genotypes have no whole genome sequence information available, this epitope is 100% identical in 256 of 258 sequences in GenBank from encompassing the years 1950–2023. The epitope sequence from two strains, Uvira B53 (Genotype X; 2019; MT956648) and BUR/18/Rutana (Genotype X; 2018; MW856067) varied by one amino acid (Q124E; peptide Q14E). In addition, the epitope recognized by F88ASF-55 cannot be found in any other organism.

### Double-antibody sandwich ELISA for ASFV detection

**Figure 4:**
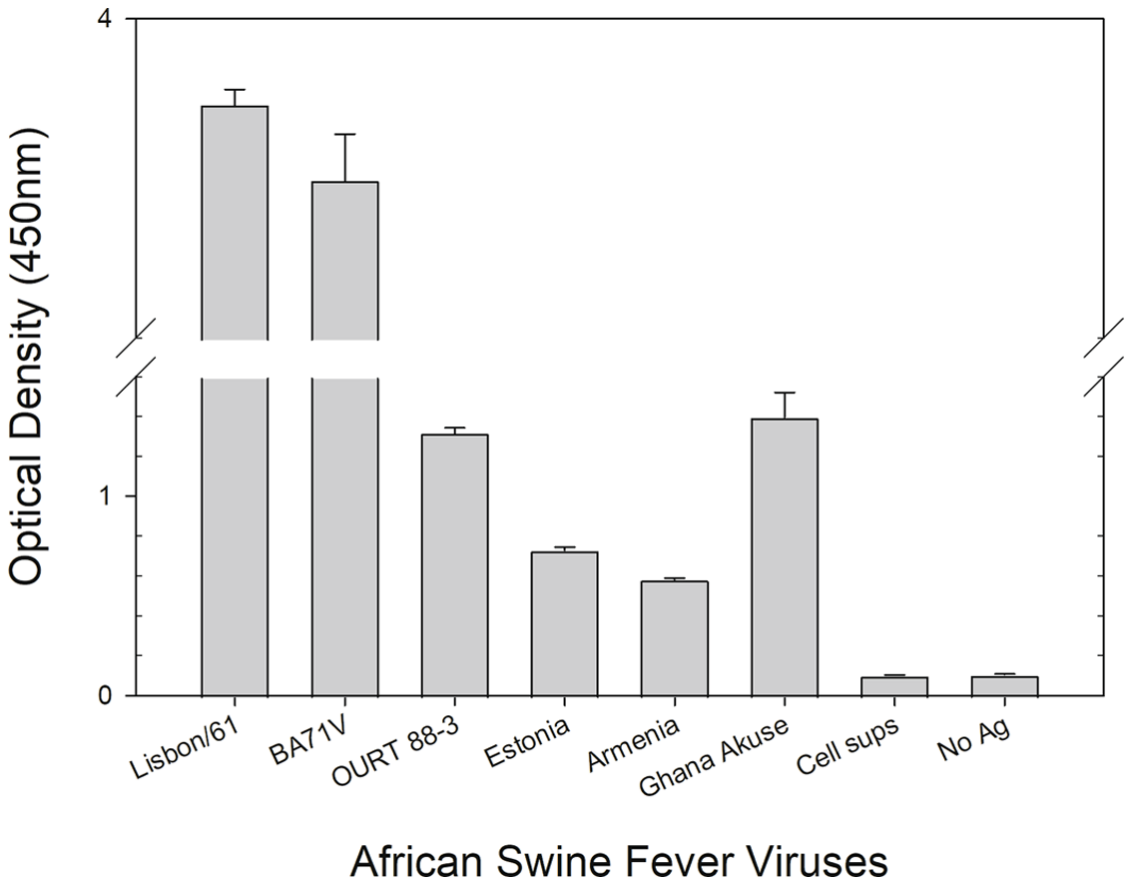
DAS ELISA for the detection of the African swine virus. In the ELISA, polyclonal anti-ASFV antibody was coated onto microtiter plates. The viruses in cell culture supernatants (Lisbon/61, BA71V, OURT 88/3, Estonia 2014, Armenia/07, and Ghana Akuse/2020) were added to the plates. Then hybridoma culture supernatant (F88ASF-55) was added. The antibodies’ binding was detected with a rabbit anti-mouse IgG-HRP conjugate. Optical density (OD) values are mean duplicates.

A DAS ELISA was used to determine whether the mAb could detect ASFV. In the ELISA, polyclonal porcine anti-ASFV was used as capture agent, and mAb F88ASF-55 was used as detection agent. The DAS ELISA identified 5 strains of ASFV genotypes I and II viruses in addition to Lisbon/61 (Figure 4). The ELISA is expected to be specific for ASFV, as cell culture supernatants and virus-free controls showed a very low background. Not all ASFV genotypes were tested due to limited sequence information available for other genotypes. F88ASF-55 has the potential to detect numerous ASFV genotypes in ELISA because this mAb targets a conserved epitope shared among ASFV genotypes with *A137R* sequences, as shown in Table 2. However, a full evaluation is required.

### ASFV antigen detection using immunohistochemistry and *in situ* hybridization

**Figure 5:**
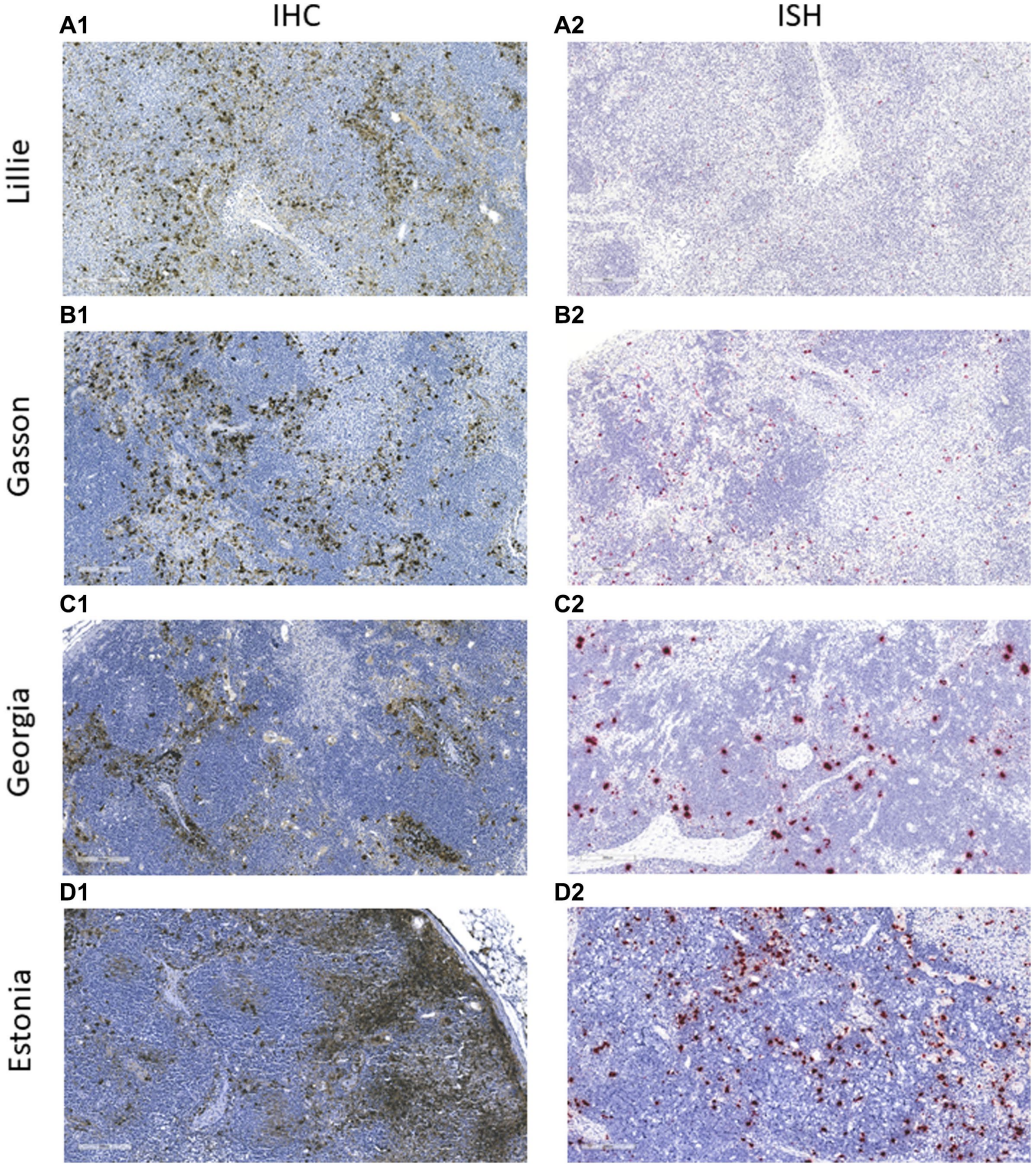
Immunohistochemistry (IHC) and *in situ* hybridization (ISH) analysis of ASFV-infected tissues. Submandibular lymph nodes from animals experimentally infected with ASFV strains **(A)** Lillie SI-85; **(B)** Gasson; **(C)** Georgia 2007/1, and **(D)** Estonia 2014 were paraffin-embedded and analyzed using IHC and ISH. In IHC, the ASFV antigen was detected using mAb F88ASF-55 **(A1–D1)**. In ISH, ASFV RNA was detected using the V-AFSV-01 probe **(A2–D2)**. The IHC and ISH negative controls are not shown.

The mAb was used in the IHC assay to detect ASFV in infected porcine tissues. IHC results indicated that F88ASF-55 was able to specifically recognize the viral antigen with low non-specific background staining in submandibular lymph nodes from animals experimentally infected with ASFV (Figure 5). The immunostaining observed was intense and specific with an excellent signal-to-noise ratio. The mAb showed no cross-reactivity with negative control tissue (data not shown).

To confirm the specificity of the IHC assay results, an ISH was performed in parallel using the same tissues (Figure 5). ASFV RNA could be detected with a similar distribution to IHC assay results, indicating that the IHC assay using F88ASF-55 detected specific ASFV antigen. However, IHC detection showed stronger signals than ISH for the four ASFV strains tested. Viral antigen concentrations in tissues at 5–10 dpi may be higher than viral RNA detected by ISH.

## Discussion

African swine fever outbreaks have occurred in major swine-producing countries in Europe, in addition to China and Mongolia recently. Thus, ASFV poses the most significant threat to the global swine industry. The rapid and early detection of ASFV infection is one of the key components in controlling this disease. In order to develop valuable immunoassays for ASFV detection, an ASFV-specific mAb was produced. The mAb binding epitope was characterized, and the diagnostic applications of the mAb were evaluated.

It has been previously reported that ASFV-specific monoclonal antibodies were produced by mice immunized with recombinant proteins (11–15), and mAbs are only highly specific for a single protein. In our laboratory, we produced mAbs from mice immunized with recombinant ASV p534, p72, and p30, however these mAbs did not react with native ASF particles (unpublished data). One possible explanation is that although the recombinant protein has the correct sequence, the protein fold may be different from the native virus, so the monoclonal antibody cannot bind to the viral particles. Therefore, these mAbs cannot be used in immunoassays for virus detection. To overcome this problem, in this study, mAbs were generated from mice immunized with ASF virus particles. One mAb F88ASF-55 was shown to react with ASFV only, without cross-reaction to unrelated recombinant classical swine fever E2 protein, and foot-and-mouth disease virus (FMDV). It has been reported that p30 is immunogenic and stimulates the highest viral antibody response during ASFV infection (20). However, mAb F88ASF-55 did not respond to p30 and other major highly immunogenic structural proteins such as p54 and p72. Despite the use of historical genotype I ASFV Lisbon/61 isolate, the generated mAb F88ASF-55 recognized different ASFV genotypes and isolates. Unusually, only one ASFV-specific mAb was finally obtained after several attempts. The reason that only one ASFV-specific mAb was yielded from the fusions may be due to the hybridoma screening method we used. First, since the mice are immunized with virus particles, it is best to use whole virus as an antigen for screening, but purified ASFV was not available in the laboratory. Second, unpurified polyclonal ASFV-positive serum (48 dpi) was used as the capture agent, which induced non-specific binding. Third, in the first screen, no-antigen negative controls were not included, so it was impossible to distinguish whether the mAb binding was to ASFV or polyserum, and thus most of the selected clones were false positives for ASFV.

Since ASFV consists of more than 50 polypeptides (21, 22), it was necessary to determine the binding protein and epitope recognized by F88ASF-55. A proteomic approach was performed to identify the binding protein. LC-MS/MS results confirmed that the epitope recognized by F88ASF-55 is an ASFV structural protein encoded by *A137R* (pA137R). Given the high expression level of the *A137R*, it may be important throughout infection, making it an interesting candidate for detection (23). Recently, ASFV pA137R was reported to inhibit the production of type-I interferon (24). Furthermore, attenuation of ASFV by *A137R* deletion in an ASFV pandemic strain offered protection against the virulent parental strain (25). In this context, pA137R is potentially a target antigen for ASF diagnosis.

The epitope bound by F88ASF-55 is linear as the mAb reacted with pA137R under the denaturing conditions of SDS-PAGE as determined by Western blot analysis. The epitope recognized by F88ASF-55 was elucidated using 14 overlapping peptides representing the whole polypeptide of ASFV pA137R. F88ASF-55 reacted with peptide #12, corresponding to amino acids 111–125 (VDFQANIENMDDLQK). The results showed that the mAb-binding epitopes of ASFV isolates from 1950–2008 were 100% identical across ASFV genotypes with available *A137R* sequences. This finding is consistent with the report by Yin et al. (23). They developed an RT-PCR assay targeting *A137R* based on the alignment of 100 complete coding sequences available in GenBank. Although the overall genome mutation rate of ASFV is relatively low, ASFV still shows genetic and antigenic diversity. Multiple base-pair mismatches have been reported when using WOAH-recommended primers and probe targeting p72, highlighting the need to monitor circulating strains for diagnostic relevance (26). As demonstrated by Yin et al. (23), real-time PCR detection of *A137R* performed better than the WOAH-recommended RT-PCR assay targeting p72 for ASFV detection in clinical samples (23). Recently, a published report described a new indirect serological ELISA using recombinant p11.5 (pA137R) protein as an antigen (27). It is supported that pA137R is a good target for diagnostic assays to detect ASFV infection.

Due to immunoassays’ robustness and reduced cost, ELISA remains one of the main platforms used for virus detection. Nonetheless, its use has drawbacks, such as antibodies cross-reacting with other co-infecting viruses, leading to false positives or inaccurate results. The use of mAbs could improve the specificity of the ELISA. Hutchings and Ferri reported a sandwich ELISA using a mAb against p72, and rabbit polyclonal serum for antigenic detection of ASFV (28). However, the report indicated the ELISA test was not as sensitive as the haemadsorption test and may not detect low concentrations of ASFV. Using mAb F88ASF-55, which recognizes pA137R but not p72, the sensitivity of the ELISA may be higher than previously reported results. Although the *A137R* sequence from numerous genotypes remains unknown, based on current sequences in GenBank, F88ASF-55 binds to a highly conserved epitope across a large temporal range (1950–2008) and at least 4 genotypes, so it may detect novel viral isolates. Since the current study aimed to demonstrate that this mAb can be used for ASFV antigen detection in ELISAs, the diagnostic specificity and sensitivity of the antigen detection ELISA has not been established. Full validation of the ELISA will be performed and published in a separate report.

IHC and ISH analyses are commonly used for the identification of specific viruses. Both technologies make it possible to visualize the distribution and localization of the virus in infected cells or tissues. Several researchers report using IHC with polyclonal antibodies for ASFV detection (12, 13). Polyclonal antibodies can have high background and low specificity, so a mAb would be preferred. A mAb against ASFV p30 was previously generated for IHC use (11). Isolates from Eastern and Southern African countries can have variability in the antigenic region of p30. Due to this variability between different ASFV isolates, p30-specific mAbs may fail to detect viral antigen, especially isolates with different geographic origins (29). Another report detected ASFV genotype I using IHC with a mAb against ASFV p72 (15), but the exact mAb-binding epitope was not identified. Since ASFV is classified into 24 genotypes based on B646L which encodes p72 (5, 30), mAbs generated to one genotype may not detect other p72 from other genotypes. It was reported that mAbs against pB602L, a non-structural protein, could detect ASFV in ASFV-infected tissue using IHC, however its performance was only evaluated with one strain (31). Although several of these mAbs worked well in IHC, more mAbs available for IHC would allow for more reliable ASFV detection. In this study, the mAb (F88-ASF55) detected ASF viral antigen in infected tissueswith no non-specific background staining in IHC assays. Although not all 24 ASFV genotypes were examined in this study, multiple different strains of genotype II viruses were detectable in both IHC assays and ELISAs using this mAb. This is important considering that genotype II ASFV has now been reported in Europe, the Russian Federation, Southeast Asia, the Dominican Republic, and Haiti, as well as Nigeria (1, 8–10). This mAb might detect all ASFV genotypes based on the recognized epitope being highly conserved. We observed more abundant staining depending on the ASFV strain, most likely reflecting the viral load, which could be due to a difference in virulence, susceptibility of individual animals, or date of sampling.

ASFV in tissues from pigs infected with the highly virulent Malawi isolate of ASFV, was principally detected in cells of the mononuclear phagocytic system when using IHC and ISH (30). In our report, ASFV RNA detected by ISH had a similar distribution of ASFV antigens as detected by IHC, suggesting that both assays can be successfully used as diagnostic tests. However, the IHC assay was considerably easier to perform, taking about 4 h to complete, compared to non-radioactive ISH, which takes 2 days (30). Interestingly, the IHC assay appeared to be more sensitive for all tested strains, detecting more viral antigen than RNA in the tissues. This may be related to the infection duration, reflecting an accumulation of viral antigen compared to viral mRNA. However, given the range of time points from 5–10 dpi, it is more likely that the IHC assay has a higher sensitivity than the ISH assay. As it is difficult to obtain or produce large numbers of positive samples for multiple different strains, were unable to evaluate diagnostic sensitivity and specificity at this time. A full evaluation is required for both approaches before we draw a final conclusion.

## Conclusion

In summary, a mAb (F88ASF-55) against ASFV was generated and characterized. This mAb recognizes the pA137R, and the epitope has been mapped using a peptide array. We identified that F88ASF-55 binds to a highly conserved epitope, which is important in diagnostic applications. The results indicate that F88ASF-55 could be used in ELISAs and IHC assays for ASFV antigen detection and possibly in immunochromatographic strip assays.

## Data availability statement

The data presented in the study are deposited at https://massive.ucsd.edu, accession numbers MassIVE MSV000092995 and ProteomeXchange PXD045773.

## Ethics statement

All archive tissues used in this study were obtained from experiments conducted according to the guidelines of the Canadian Council for Animal Care. All experiments were approved by the institutional animal care committee and performed at the National Centre for Foreign Animal Disease.

## Author contributions

CE-H: Data curation, Formal analysis, Investigation, Methodology, Supervision, Writing – original draft, Writing – review & editing. EM: Data curation, Methodology, Writing – review & editing. DZ: Data curation, Methodology, Writing – review & editing. CE: Data curation, Formal analysis, Methodology, Validation, Writing – review & editing. BC: Formal analysis, Methodology, Writing – review & editing. KG: Writing – original draft. AA: Funding acquisition, Project administration, Resources, Supervision, Writing – review & editing. MY: Conceptualization, Data curation, Formal analysis, Funding acquisition, Investigation, Project administration, Resources, Supervision, Validation, Writing – original draft, Writing – review & editing.
